# Supplementary material for: COZOID: contact zone identifier for visual analysis of protein-protein interactions
Source: BMC Bioinformatics. 2018 Apr 6;19:125. doi: 10.1186/s12859-018-2113-6 (PMC5889581; doi:10.1186/s12859-018-2113-6)
Supplement: Supplementary file 2 — Software build. Executable binary file of the software tool. (ZIP 118,784 kb) [file 12859_2018_2113_MOESM2_ESM.zip › cozoid/java/docs/beansbinding-1.2.1-doc.zip!/org/jdesktop/el/ELResolver.html]

ELResolver (Beans Binding)


---


|  |  |  |
| --- | --- | --- |
| **Overview** \| **Package** \| **Class** \| **Use** \| **Tree** \| **Deprecated** \| **Index** \| **Help** | |  |
| **PREV CLASS**   **NEXT CLASS** | **FRAMES**    **NO FRAMES**     **All Classes** |  |
| SUMMARY: NESTED \| FIELD \| CONSTR \| METHOD | DETAIL: FIELD \| CONSTR \| METHOD |  |


---


## org.jdesktop.el Class ELResolver

```
java.lang.Object
  org.jdesktop.el.ELResolver
```

**Direct Known Subclasses:**: ArrayELResolver, BeanELResolver, CompositeELResolver, ListELResolver, MapELResolver, ResourceBundleELResolver

---

``` public abstract class ELResolver extends java.lang.Object ```

Enables customization of variable and property resolution behavior for EL
expression evaluation.

While evaluating an expression, the `ELResolver` associated
with the `ELContext` is consulted to do the initial resolution of
the first variable of an expression. It is also consulted when a
`.` or `[]` operator is encountered, except for the
last such operator in a method expression, in which case the resultion
rules are hard coded.

For example, in the EL expression `${employee.lastName}`,
the `ELResolver` determines what object `employee`
refers to, and what it means to get the `lastName` property on
that object.

Most methods in this class accept a `base`
and `property` parameter. In the case of variable resolution
(e.g. determining what `employee` refers to in
`${employee.lastName}`), the `base` parameter will
be `null` and the `property` parameter will always
be of type `String`. In this case, if the `property`
is not a `String`, the behavior of the `ELResolver`
is undefined.

In the case of property resolution, the `base` parameter
identifies the base object and the `property` object identifies
the property on that base. For example, in the expression
`${employee.lastName}`, `base` is the result of the
variable resolution for `employee` and `property`
is the string `"lastName"`. In the expression
`${y[x]}`, `base` is the result of the variable
resolution for `y` and `property` is the result of
the variable resolution for `x`.

Though only a single `ELResolver` is associated with an
`ELContext`, there are usually multiple resolvers considered
for any given variable or property resolution. `ELResolver`s
are combined together using `CompositeELResolver`s, to define
rich semantics for evaluating an expression.

For the `getValue(org.jdesktop.el.ELContext, java.lang.Object, java.lang.Object)`, `getType(org.jdesktop.el.ELContext, java.lang.Object, java.lang.Object)`, `setValue(org.jdesktop.el.ELContext, java.lang.Object, java.lang.Object, java.lang.Object)` and
`isReadOnly(org.jdesktop.el.ELContext, java.lang.Object, java.lang.Object)` methods, an `ELResolver` is not
responsible for resolving all possible (base, property) pairs. In fact,
most resolvers will only handle a `base` of a single type.
To indicate that a resolver has successfully resolved a particular
(base, property) pair, it must set the `propertyResolved`
property of the `ELContext` to `true`. If it could
not handle the given pair, it must leave this property alone. The caller
must ignore the return value of the method if `propertyResolved`
is `false`.

The `getFeatureDescriptors(org.jdesktop.el.ELContext, java.lang.Object)` and `getCommonPropertyType(org.jdesktop.el.ELContext, java.lang.Object)`
methods are primarily designed for design-time tool support, but must
handle invocation at runtime as well. The
`Beans.isDesignTime()` method can be used to determine
if the resolver is being consulted at design-time or runtime.

**Since:**
:   JSP 2.1

**See Also:**: `CompositeELResolver`, `ELContext.getELResolver()`

---

| **Field Summary** | |
| --- | --- |
| `static java.lang.String` | `RESOLVABLE_AT_DESIGN_TIME`             The attribute name of the named attribute in the `FeatureDescriptor` that specifies whether the variable or property can be resolved at runtime. |
| `static java.lang.String` | `TYPE`             The attribute name of the named attribute in the `FeatureDescriptor` that specifies the runtime type of the variable or property. |


**Constructor Summary**

`ELResolver()`


| **Method Summary** | |
| --- | --- |
| `abstract  java.lang.Class<?>` | `getCommonPropertyType(ELContext context, java.lang.Object base)`             Returns the most general type that this resolver accepts for the `property` argument, given a `base` object. |
| `abstract  java.util.Iterator<java.beans.FeatureDescriptor>` | `getFeatureDescriptors(ELContext context, java.lang.Object base)`             Returns information about the set of variables or properties that can be resolved for the given `base` object. |
| `abstract  java.lang.Class<?>` | `getType(ELContext context, java.lang.Object base, java.lang.Object property)`             For a given `base` and `property`, attempts to identify the most general type that is acceptable for an object to be passed as the `value` parameter in a future call to the `setValue(org.jdesktop.el.ELContext, java.lang.Object, java.lang.Object, java.lang.Object)` method. |
| `abstract  java.lang.Object` | `getValue(ELContext context, java.lang.Object base, java.lang.Object property)`             Attempts to resolve the given `property` object on the given `base` object. |
| `abstract  boolean` | `isReadOnly(ELContext context, java.lang.Object base, java.lang.Object property)`             For a given `base` and `property`, attempts to determine whether a call to `setValue(org.jdesktop.el.ELContext, java.lang.Object, java.lang.Object, java.lang.Object)` will always fail. |
| `abstract  void` | `setValue(ELContext context, java.lang.Object base, java.lang.Object property, java.lang.Object value)`             Attempts to set the value of the given `property` object on the given `base` object. |

**Methods inherited from class java.lang.Object**

`clone, equals, finalize, getClass, hashCode, notify, notifyAll, toString, wait, wait, wait`

**Field Detail**

### TYPE

```
public static final java.lang.String TYPE
```

:   The attribute name of the named attribute in the
    `FeatureDescriptor` that specifies the runtime type of
    the variable or property.

    **See Also:**: Constant Field Values

---


### RESOLVABLE\_AT\_DESIGN\_TIME

```
public static final java.lang.String RESOLVABLE_AT_DESIGN_TIME
```

:   The attribute name of the named attribute in the
    `FeatureDescriptor` that specifies whether the
    variable or property can be resolved at runtime.

    **See Also:**: Constant Field Values


**Constructor Detail**

### ELResolver

```
public ELResolver()
```


**Method Detail**

### getValue

```
public abstract java.lang.Object getValue(ELContext context,
                                          java.lang.Object base,
                                          java.lang.Object property)
```

:   Attempts to resolve the given `property` object on the given
    `base` object.

    If this resolver handles the given (base, property) pair,
    the `propertyResolved` property of the
    `ELContext` object must be set to `true`
    by the resolver, before returning. If this property is not
    `true` after this method is called, the caller should ignore
    the return value.

    :   **Parameters:**: `context` - The context of this evaluation.: `base` - The base object whose property value is to be returned, or `null` to resolve a top-level variable.: `property` - The property or variable to be resolved. **Returns:**: If the `propertyResolved` property of `ELContext` was set to `true`, then the result of the variable or property resolution; otherwise undefined. **Throws:**: `java.lang.NullPointerException` - if context is `null`: `PropertyNotFoundException` - if the given (base, property) pair is handled by this `ELResolver` but the specified variable or property does not exist or is not readable.: `ELException` - if an exception was thrown while performing the property or variable resolution. The thrown exception must be included as the cause property of this exception, if available.

---


### getType

```
public abstract java.lang.Class<?> getType(ELContext context,
                                           java.lang.Object base,
                                           java.lang.Object property)
```

:   For a given `base` and `property`, attempts to
    identify the most general type that is acceptable for an object to be
    passed as the `value` parameter in a future call
    to the `setValue(org.jdesktop.el.ELContext, java.lang.Object, java.lang.Object, java.lang.Object)` method.

    If this resolver handles the given (base, property) pair,
    the `propertyResolved` property of the
    `ELContext` object must be set to `true`
    by the resolver, before returning. If this property is not
    `true` after this method is called, the caller should ignore
    the return value.

    This is not always the same as `getValue().getClass()`.
    For example, in the case of an `ArrayELResolver`, the
    `getType` method will return the element type of the
    array, which might be a superclass of the type of the actual
    element that is currently in the specified array element.

    :   **Parameters:**: `context` - The context of this evaluation.: `base` - The base object whose property value is to be analyzed, or `null` to analyze a top-level variable.: `property` - The property or variable to return the acceptable type for. **Returns:**: If the `propertyResolved` property of `ELContext` was set to `true`, then the most general acceptable type; otherwise undefined. **Throws:**: `java.lang.NullPointerException` - if context is `null`: `PropertyNotFoundException` - if the given (base, property) pair is handled by this `ELResolver` but the specified variable or property does not exist or is not readable.: `ELException` - if an exception was thrown while performing the property or variable resolution. The thrown exception must be included as the cause property of this exception, if available.

---


### setValue

```
public abstract void setValue(ELContext context,
                              java.lang.Object base,
                              java.lang.Object property,
                              java.lang.Object value)
```

:   Attempts to set the value of the given `property`
    object on the given `base` object.

    If this resolver handles the given (base, property) pair,
    the `propertyResolved` property of the
    `ELContext` object must be set to `true`
    by the resolver, before returning. If this property is not
    `true` after this method is called, the caller can
    safely assume no value has been set.

    :   **Parameters:**: `context` - The context of this evaluation.: `base` - The base object whose property value is to be set, or `null` to set a top-level variable.: `property` - The property or variable to be set.: `value` - The value to set the property or variable to. **Throws:**: `java.lang.NullPointerException` - if context is `null`: `PropertyNotFoundException` - if the given (base, property) pair is handled by this `ELResolver` but the specified variable or property does not exist.: `PropertyNotWritableException` - if the given (base, property) pair is handled by this `ELResolver` but the specified variable or property is not writable.: `ELException` - if an exception was thrown while attempting to set the property or variable. The thrown exception must be included as the cause property of this exception, if available.

---


### isReadOnly

```
public abstract boolean isReadOnly(ELContext context,
                                   java.lang.Object base,
                                   java.lang.Object property)
```

:   For a given `base` and `property`, attempts to
    determine whether a call to `setValue(org.jdesktop.el.ELContext, java.lang.Object, java.lang.Object, java.lang.Object)` will always fail.

    If this resolver handles the given (base, property) pair,
    the `propertyResolved` property of the
    `ELContext` object must be set to `true`
    by the resolver, before returning. If this property is not
    `true` after this method is called, the caller should ignore
    the return value.

    :   **Parameters:**: `context` - The context of this evaluation.: `base` - The base object whose property value is to be analyzed, or `null` to analyze a top-level variable.: `property` - The property or variable to return the read-only status for. **Returns:**: If the `propertyResolved` property of `ELContext` was set to `true`, then `true` if the property is read-only or `false` if not; otherwise undefined. **Throws:**: `java.lang.NullPointerException` - if context is `null`: `PropertyNotFoundException` - if the given (base, property) pair is handled by this `ELResolver` but the specified variable or property does not exist.: `ELException` - if an exception was thrown while performing the property or variable resolution. The thrown exception must be included as the cause property of this exception, if available.

---


### getFeatureDescriptors

```
public abstract java.util.Iterator<java.beans.FeatureDescriptor> getFeatureDescriptors(ELContext context,
                                                                                       java.lang.Object base)
```

:   Returns information about the set of variables or properties that
    can be resolved for the given `base` object. One use for
    this method is to assist tools in auto-completion.

    If the `base` parameter is `null`, the
    resolver must enumerate the list of top-level variables it can
    resolve.

    The `Iterator` returned must contain zero or more
    instances of `FeatureDescriptor`, in no guaranteed
    order. In the case of primitive types such as `int`, the
    value `null` must be returned. This is to prevent the
    useless iteration through all possible primitive values. A
    return value of `null` indicates that this resolver does
    not handle the given `base` object or that the results
    are too complex to represent with this method and the
    `getCommonPropertyType(org.jdesktop.el.ELContext, java.lang.Object)` method should be used instead.

    Each `FeatureDescriptor` will contain information about
    a single variable or property. In addition to the standard
    properties, the `FeatureDescriptor` must have two
    named attributes (as set by the `setValue` method):

    - `TYPE` - The value of this named attribute must be
      an instance of `java.lang.Class` and specify the
      runtime type of the variable or property.
    - `RESOLVABLE_AT_DESIGN_TIME` - The value of this
      named attribute must be an instance of
      `java.lang.Boolean` and indicates whether it is safe
      to attempt to resolve this property at design-time. For
      instance, it may be unsafe to attempt a resolution at design
      time if the `ELResolver` needs access to a resource
      that is only available at runtime and no acceptable simulated
      value can be provided.

    The caller should be aware that the `Iterator`
    returned might iterate through a very large or even infinitely large
    set of properties. Care should be taken by the caller to not get
    stuck in an infinite loop.

    This is a "best-effort" list. Not all `ELResolver`s
    will return completely accurate results, but all must be callable
    at both design-time and runtime (i.e. whether or not
    `Beans.isDesignTime()` returns `true`),
    without causing errors.

    The `propertyResolved` property of the
    `ELContext` is not relevant to this method.
    The results of all `ELResolver`s are concatenated
    in the case of composite resolvers.

    :   **Parameters:**: `context` - The context of this evaluation.: `base` - The base object whose set of valid properties is to be enumerated, or `null` to enumerate the set of top-level variables that this resolver can evaluate. **Returns:**: An `Iterator` containing zero or more (possibly infinitely more) `FeatureDescriptor` objects, or `null` if this resolver does not handle the given `base` object or that the results are too complex to represent with this method **See Also:**: `FeatureDescriptor`

---


### getCommonPropertyType

```
public abstract java.lang.Class<?> getCommonPropertyType(ELContext context,
                                                         java.lang.Object base)
```

:   Returns the most general type that this resolver accepts for the
    `property` argument, given a `base` object.
    One use for this method is to assist tools in auto-completion.

    This assists tools in auto-completion and also provides a
    way to express that the resolver accepts a primitive value,
    such as an integer index into an array. For example, the
    `ArrayELResolver` will accept any `int` as a
    `property`, so the return value would be
    `Integer.class`.

    :   **Parameters:**: `context` - The context of this evaluation.: `base` - The base object to return the most general property type for, or `null` to enumerate the set of top-level variables that this resolver can evaluate. **Returns:**: `null` if this `ELResolver` does not know how to handle the given `base` object; otherwise `Object.class` if any type of `property` is accepted; otherwise the most general `property` type accepted for the given `base`.


---


|  |  |  |
| --- | --- | --- |
| **Overview** \| **Package** \| **Class** \| **Use** \| **Tree** \| **Deprecated** \| **Index** \| **Help** | |  |
| **PREV CLASS**   **NEXT CLASS** | **FRAMES**    **NO FRAMES**     **All Classes** |  |
| SUMMARY: NESTED \| FIELD \| CONSTR \| METHOD | DETAIL: FIELD \| CONSTR \| METHOD |  |


---
